# Heteromorphic variants of chromosome 9

**DOI:** 10.1186/1755-8166-6-14

**Published:** 2013-04-01

**Authors:** Nadezda Kosyakova, Ani Grigorian, Thomas Liehr, Marina Manvelyan, Isabella Simonyan, Hasmik Mkrtchyan, Rouben Aroutiounian, Anna D Polityko, Anna I Kulpanovich, Tatiana Egorova, Evgenia Jaroshevich, Alla Frolova, Natalia Shorokh, Irina V Naumchik, Marianne Volleth, Isolde Schreyer, Heike Nelle, Markus Stumm, Rolf-Dieter Wegner, Gisela Reising-Ackermann, Martina Merkas, Lukretija Brecevic, Thomas Martin, Laura Rodríguez, Samarth Bhatt, Monika Ziegler, Katharina Kreskowski, Anja Weise, Ali Sazci, Svetlana Vorsanova, Marcelo de Bello Cioffi, Emel Ergul

**Affiliations:** 1 Jena University Hospital, Friedrich Schiller University, Institute of Human Genetics, Kollegiengasse 10, D-07743, Jena, Germany; 2 Research Center of Maternal and Child Health Protection, Mashtots Ave. 22, 0002, Yerevan, Armenia; 3 Centre of Medical Genetics and Primary Health Care, Abovyan av. 34/3, Yerevan, Armenia; 4 Department of Genetic and Laboratory of Cytogenetics, State University, 1, Alex Manoukian Street, Yerevan, Armenia; 5 National Medical Center ‘Mother and Child’, Orlovskaya Str. 66, 220053, Minsk, Belarus; 6 Regional Medical Genetics Center, Kirova str, 57, 246022, Gomel, Belarus; 7 Regional Medical Genetics Center, Kirova str., 88, 224013, Brest, Belarus; 8 Institute of Human Genetics, Leipziger Str. 44, 39120, Magdeburg, Germany; 9 Zentrum für Ambulante Medizin, Jena University Hospital, Carl Zeiß-Platz 8 07743, Jena, Germany; 10 Children Hospital, Jena University Hospital, Friedrich Schiller University, Kochstr. 2, D-07743, Jena, Germany; 11 Partnerschaft, Kurfürstendamm, 19910719, Berlin, Germany; 12 MVZ-Labor, Strümpellstr. 40, 04289, Leipzig, Germany; 13 School of Medicine Zagreb University, Croatian Institute for Brain Research, Zagreb, Croatia; 14 Genetische Beratung und klinische Genetik Biomedizinisches Zentrum, Kardinal-Wendel-Str. 14, 66424, Homburg, Germany; 15 AbaCid-Genética Hospital de Madrid Norte Sanchinarro, Madrid, Spain; 16 Department of Medical Biology and Genetics, Faculty of Medicine, University of Kocaeli, Kocaeli, Turkey; 17 Institute of Pediatrics and Children Surgery, Ministry of Health of the Russian Federation, 125412, Moscow, Russia; 18 Departamento de Genetica e Evolucao, Universidade Federal de Sao Carlos, Sao Carlos, SP, Brazil

**Keywords:** Chromosome 9, Heteromorphism, Breakpoints, Western Europe, Eastern Europe

## Abstract

**Background:**

Heterochromatic variants of pericentromere of chromosome 9 are reported and discussed since decades concerning their detailed structure and clinical meaning. However, detailed studies are scarce. Thus, here we provide the largest ever done molecular cytogenetic research based on >300 chromosome 9 heteromorphism carriers.

**Results:**

In this study, 334 carriers of heterochromatic variants of chromosome 9 were included, being 192 patients from Western Europe and the remainder from Easter-European origin. A 3-color-fluorescence in situ hybridization (FISH) probe-set directed against for 9p12 to 9q13~21.1 (9het-mix) and 8 different locus-specific probes were applied for their characterization. The 9het-mix enables the characterization of 21 of the yet known 24 chromosome 9 heteromorphic patterns. In this study, 17 different variants were detected including five yet unreported; the most frequent were pericentric inversions (49.4%) followed by 9qh-variants (23.9%), variants of 9ph (11.4%), cenh (8.2%), and dicentric- (3.8%) and duplication-variants (3.3%). For reasons of simplicity, a new short nomenclature for the yet reported 24 heteromorphic patterns of chromosome 9 is suggested. Six breakpoints involved in four of the 24 variants could be narrowed down using locus-specific probes.

**Conclusions:**

Based on this largest study ever done in carriers of chromosome 9 heteromorphisms, three of the 24 detailed variants were more frequently observed in Western than in Eastern Europe. Besides, there is no clear evidence that infertility is linked to any of the 24 chromosome 9 heteromorphic variants.

## Background

Chromosome 9 presents the highest degree of morphological variations among the non-acrocentric human chromosomes. Variants include 9qh+, 9cenh+, 9ph+, 9qh-, or inv(9)(p11q13), and they are commonly found in routine cytogenetics, with an overall frequency of approximately 1.5% in the general population [1]. These variants, known as ‘heteromorphisms’, or ‘heterochromatic variants’, are usually ascertained by banding techniques in routine cytogenetics. Besides, a few molecular cytogenetic studies have been conducted for these variants [2-10]. Some studies [9,10] pointed out that large scale inherited variants almost always consist of low copy repeats or segmental duplications and that chromosome 9 is structurally highly polymorphic with a high level of intra- and interchromosomal duplications. Segmental duplications located adjacent to the centromere and to the heterochromatic block comprise ~7% of chromosome 9, and those are thought to predispose and mediate structural rearrangements within the pericentromeric region [10].

The aforementioned fluorescence in situ hybridization (FISH) studies used different probes and classified different heterochromatic variants: (i) three different types of heterochromatic patterns were reported by [2]: (ii) seven different types of rearrangements were described by [3,4]; (iii) and four different variants were identified in [10].

The clinical significance of these heteromorphisms is not well understood yet. Although, different clinical conditions associated with pericentric inversion 9 including mental retardation, schizophrenia, the Walker-Warburg syndrome, the oculo-auriculo-vertebral (Goldenhar) spectrum, cancer predisposition and infertility have been reported and discussed [6]. Recently, evidence has been provided that the presence of a constitutional inversion 9 is correlated with a significantly enhanced rate of numerical aberrations in the sperm of such carriers [11], maybe due to influences on the formation of synaptonemal complex [12]. However, contradictory studies not indicative for any adverse effect of chromosome 9 variants on fertility may be found in the literature [13]. Besides inversion, heteromorphic patterns of the pericentric chromosome 9 region were also reported to be possibly associated with infertility, especially in male [14-16].

Here we present the largest FISH-study ever done on chromosome 9 in 334 heteromorphism carriers with 423 variants. 17 different heteromorphic patterns plus a normal (i.e. the most frequent) pattern were observed and studied in detail by locus-specific probes. We have aligned the previously published and currently reported variants, and suggested a simplified nomenclature. As patients were derived from Eastern and Western Europe, the data were also analyzed concerning potential ethnic differences of heteromorphic patterns. It was also checked if carriers of chromosome 9 heteromorphisms detected due to infertility belong to any special subgroup.

## Material and methods

### Patients

The 334 carrier patients of heterochromatic variants of chromosome 9 studied here included 189 female and 139 male patients (Table 1). The gender of the remaining patients was not available/not allowed to be reported.

**Table 1:** Summary of the 334 cases with overall 423 heteromorphic variants; number of cases with any of the 17 variants detected in this study, number of male and female cases, indications, origin (Eastern or Western Europe), inheritance and presence of an additional rearrangement are listed

**Variant**	**How often variant was found**	**Female**	**Male**	**Indication and origin of carrier**			**Inheritance**			**Additional rearrangements (other than chromosome 9)**
				**indication**	**Eastern Europe**	**Western Europe**	**dn**	**mat**	**pat**	
inv(9)(var1)	101	45	48	infertility	19	16	1 on mat chr.	6	9	4
				prenatal	9	5				
				DD/ MR	11	18				
				others	4	9				
inv(9)(var2)	98	55	31	infertility	26	16	0	4	1	2
				prenatal	2	7				
				DD/ MR	7	13				
				others	2	14				
inv(9)(var2a)	9	8	1	infertility	1	0	0	2	1	1
				prenatal	1	3				
				DD/ MR	4	0				
				others	0	0				
inv(9)(het)	1	1	0	prenatal	0	1	0	1	0	0
9qh+	86	46	36	infertility	16	12	0	2	2	2
				prenatal	2	6				
				DD/ MR	5	6				
				others	5	3				
9qh+(var1)	2	0	2	prenatal	1	0	0	0	1	0
9qh-	13	28	12	infertility	7	4	0	1	1	0
				prenatal	1	3				
				DD/ MR	3	1				
				others	0	1				
9ph+	46	18	20	infertility	4	12	0	3	1	2
				prenatal	2	11				
				DD/ MR	1	8				
				others	1	1				
9ph++	1	0	1	infertility	0	1	0	0	0	0
9ph-	1	1	0	infertility	0	1	0	0	0	0
dic(9)(var1)	13	10	2	infertility	1	4	0	1	1	0
				prenatal	0	2				
				DD/ MR	0	1				
				others	0	0				
dic(9)(var2)	1	1	0	prenatal	0	1	0	0	0	0
dic(9)(var3)	2	0	2	infertility	1	0	0	0	0	0
				prenatal	0	1				
dup(9)(var1)	12	6	3	infertility	0	6	0	0	0	0
				prenatal	2	1				
				DD/ MR	0	1				
				others	1	1				
dup(9)(var2)	2	2	0	infertility	0	1	0	0	0	0
				prenatal	0	1				
9cenh+	32	16	15	infertility	6	4	0	1	0	0
				prenatal	2	6				
				DD/ MR	3	6				
				others	3	0				
9cenh-	3	0	3	prenatal	1	1	0	1	0	0
				DD/ MR	1	0				
**Sum**	**423**	**237**	**176**	infertility	**81**	**77**	**1**	**22**	**17**	**11**
				prenatal	**23**	**49**				
				DD/ MR	**35**	**54**				
				others	**16**	**29**				

E-Europe = majority: Armenia, Belarus, Turkey; single cases from: Greece, Serbia.

W-Europe = majority: Germany; single cases from: Croatia, Hungary, Spain, Switzerland.

Other aberrations: del(4)(p15.2), 45,X/46,XY/47,XY, 45,X/46,XX, del(9)(p24p22), t(4;6)(q27;p21.32), inv(7)(q35q36.1~36.2), t(15;21)(p12;q11.2~q21), der(9)t(6;9), +21, +14 and +21, t_rob_(22;22).

Abbreviations: DD = developmental delay; dn = de novo; mat = maternal, MR = mental retardation; pat = paternal.

For the study appropriate informed consent was obtained from the participating human subjects by all institutions collecting / providing the samples. The patients were acquired in different countries during routine diagnostics; all of them signed informed consent forms that they agree in the fact that research and publication is done with their samples. The German laboratory in Jena conducting all here presented FISH-experiments is certified by the German accreditation agency (Deutsche Akkreditierungsstelle GmbH = DAkkS). They approved the used forms for patient information, which includes these declarations.

Out of the total cases, 142 were derived from Eastern Europe (i.e. Armenia, Belarus and Turkey; including single cases from Greece and Serbia) and 192 from Western Europe (i.e. Germany including single cases from: Croatia, Hungary, Spain and Switzerland). A single case was from Korea. Indication for banding cytogenetic analysis was infertility (151 cases), prenatal diagnosis (55 cases), developmental delay (DD) and/or mental retardation (MR) (83 cases) or other reasons (45 cases).

Besides having heterochromatic variants on both chromosomes 9, in twenty of the 334 cases (6%), eleven cases (3.3%) had additional chromosomal aberrations, i.e. mos mos 45,X/47,XXY/46,XY; 45,X/46,XX; +21; +14 and +21; del(4)(p15.2); del(9)(p24p22); t(4;6)(q27;p21.32); inv(7)(q35q36.1~36.2); t(15;21)(p12;q11.2~q21); der(9)t(6;9); t_rob_(22;22).

### FISH-approaches

**Figure 1:**
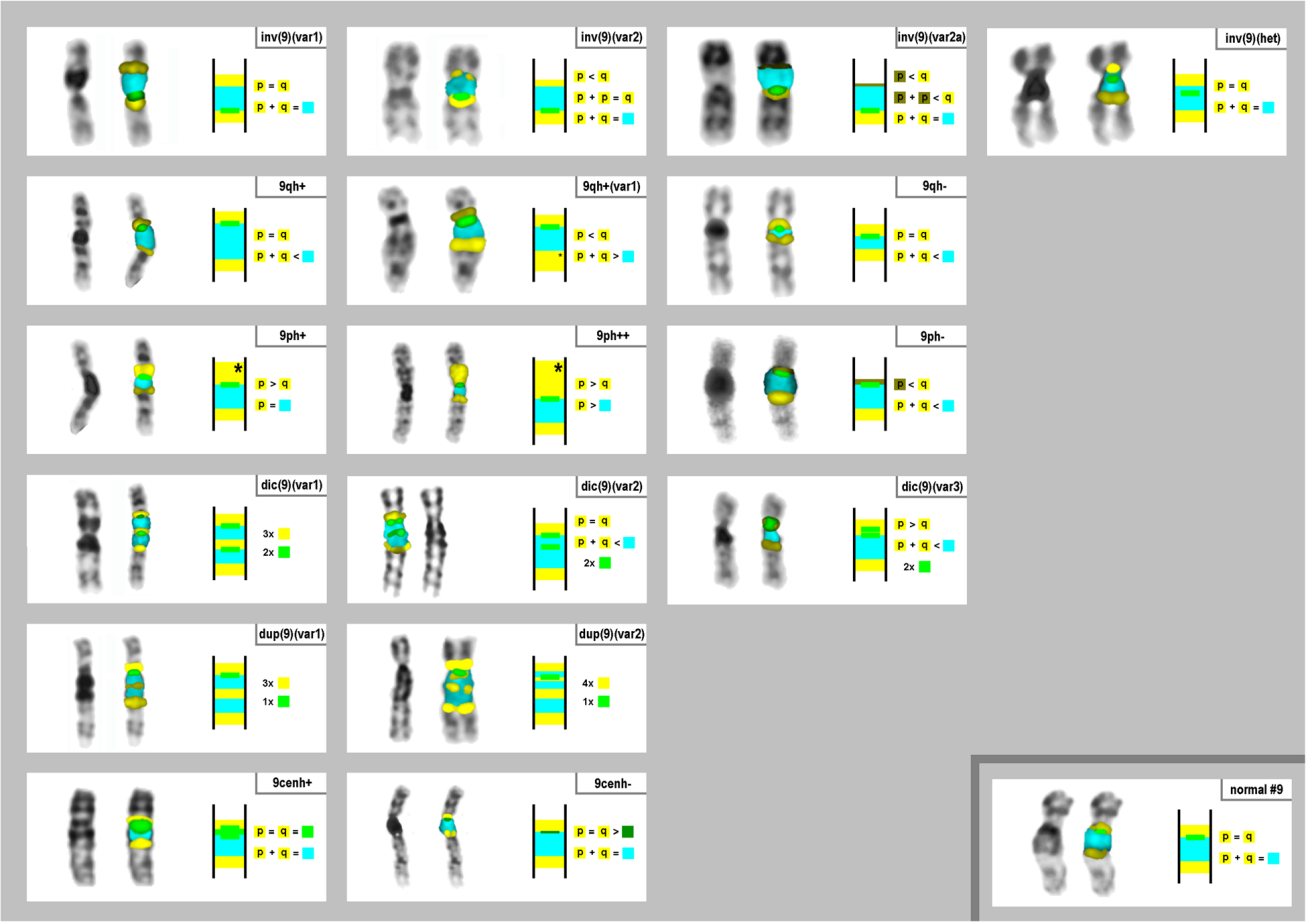
**The 17 here detected variants of chromosome 9 heteromorphisms and the normal variant (right lower edge, ‘normal #9’) are depicted in 18 frames.** Each frame shows from left to the right: inverted DAPI-banding pattern of the corresponding chromosome 9 variant, the same chromosome with FISH-signals using the 9het-mix, a schematic depiction of the FISH-result and a short description of the sizes and color intensities of the different FISH-signals. The alpha-satellite probe is labeled in green, midi18 in blue and midi36 in yellow. In the normal variant the two midi36 signals have an equal size (i.e. yellow under-laid “p = q”) and both midi36 signals have the equal size like the midi18 signal (i.e. yellow under-laid “p + q” equals the blue square). Further abbreviations of the latter short description: ‘>’ = larger in size; ‘<’ smaller in size; dark yellow or green color in square = weak signal; ‘*’ = strong signal; ‘2x’, ‘3x’ or ‘4x’= corresponding signal is present two, three or four times.

For characterization and distinguishing of the heteromorphic patterns of chromosome 9, a similar probe set was applied as reported [6] (Figure 1). It consisted of a commercially available chromosome 9 alpha-satellite probe (Abbott/ Vysis) combined with two microdissection derived probes: (i) midi36 (specific for 9p12 and 9q13~21.1) [6] and (ii) a probe specific for 9q12 (midi18) [17]. We termed the probe set as 9het-mix. The normal hybridization pattern of the 9het-mix and 12 variants of it were already reported in [6].

**Figure 2:**
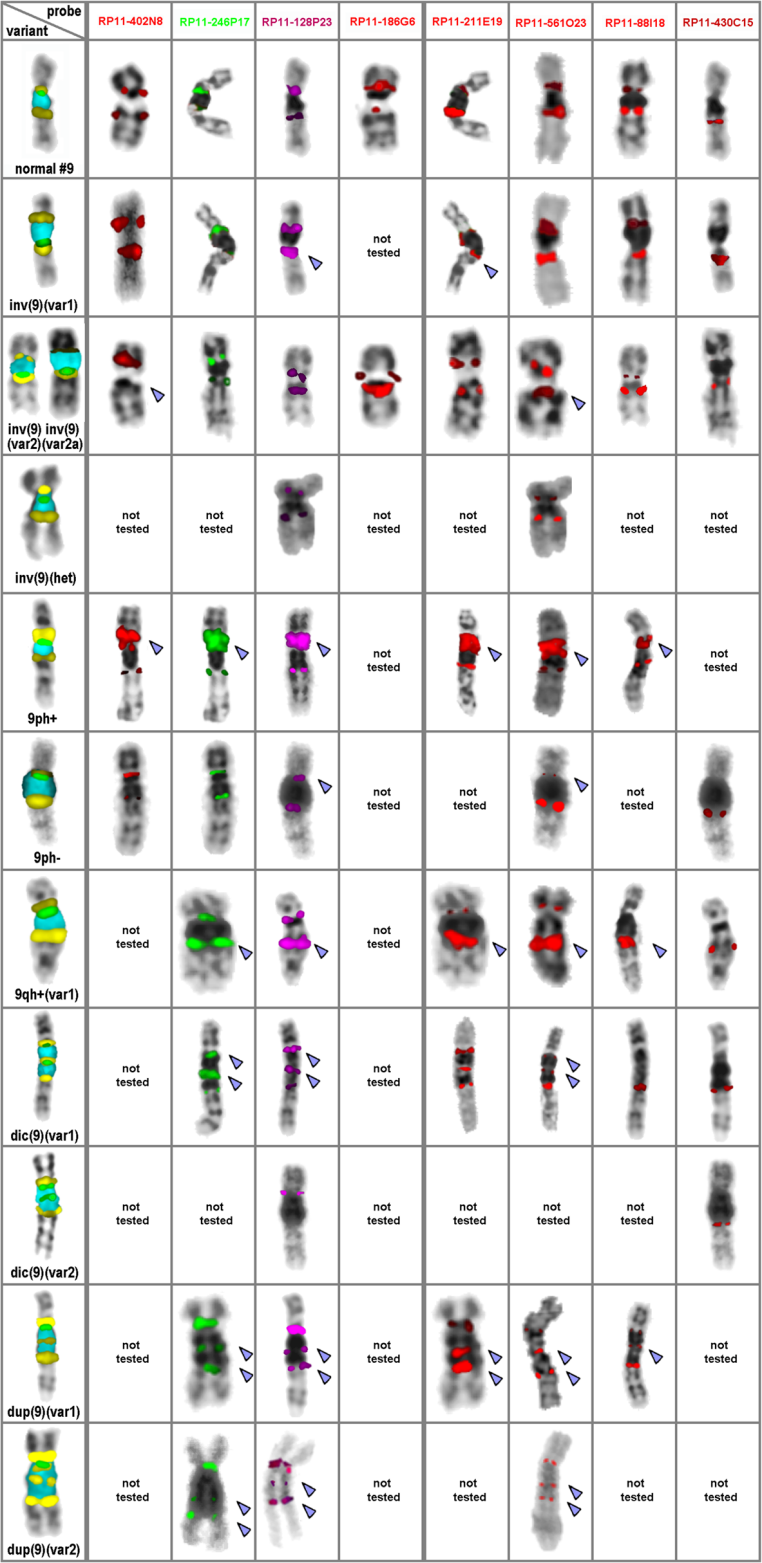
**Obtained hybridization patterns observed after application of the 8 locus-specific probes mentioned in the first row of the depiction in 11 of the 17 heteromorphic patterns of Figure 1.** Results for variants 9qh+ and 9qh- are not presented as they were similar as for the normal variant (normal #9). Breakpoints for inversions, amplification signals or duplications are highlighted by arrowheads.

Besides, eight bacterial artificial chromosome (BAC)-probes were applied for further characterization of the breakpoints of the heteromorphic variants distinguished by the 9het-mix. All BAC-probes were derived from 9p12 or 9q13~21.1; as these regions contain DNA-stretches homologous to each other cross-hybridization present for all 9p-arm probes in 9q and vice versa (Figure 2). Most of the BACs were previously published to be suited to distinguish chromosome 9 heteromorphic or other variants:

RP11-402N8 in 9p13.1 (hg19: 38,838,558-38,841,913) [8,9],

RP11-246P17 in 9p13.1 (hg19: 38,869,440-39,046,984) [9],

RP11-128P23 in 9p12 (hg19: 41,857,000-42,014,000) [18],

RP11-186G6 in 9p11.2 (hg19: 43,318,619-43,319,576),

RP11-211E19 in 9q21.11 (hg19: 70,091,000-71,043,000),

RP11-561O23 in 9q21.11 (hg19: 70,943,400-70,945,344) [9],

RP11-88I18 in 9q21.11 (hg19: 71,012,849-71,014,187) [9] and

RP11-430C15 in 9q21.11 (hg19: 71,366,061-71,568,178) [18].

Not all 17 variants characterized by the 9het-mix could be studied by all BAC-probes mainly due to lack of material. One case each was available for variations inv(9)(het), 9ph-, 9qh+, 9qh-, dup(9)(var1), dup(9)(var2), dic(9)(var1) and 9qh+(var1), three cases of variants inv(9)(var1), inv(9)(var2) and inv(9)(var2a) each plus five 9ph+ cases were studied.

For 9het-mix as well as BAC-FISH studies 10 to 20 metaphase spreads were analyzed for each case.

## Results

334 carriers of one (249 cases), two (81 cases), three (3 cases) or four (1 case) heterochromatic chromosome 9 variants were studied (Table 1 and Table 2). 43 out of the 63 cases with more than one heteromorphic variant had them on the identical chromosome; the remainder were distributed on the two homologous ones (Table 2). 423 heterochromatic variants falling into 17 subgroups (Figure 1) were detected in the studied 334 patients (Table 1).

**Table 2:** Variants going together on same chromosome (43 cases – marked with ‘sc’) and homologous chromosome (20 cases – marked with ‘hc’) are listed in detail

**Variant**	**9ph+**	**9qh+**	**9qh-**	**9cenh+**	**9cenh-**	**inv(9)(var1)**	**inv(9)(var2)**	**inv(9)(var2a)**
9ph+		2 (sc)	4 (sc)	6 (sc)		1 (sc)		
9ph+9cenh+			1 (sc)					
9qh+	1 (hc)	1 (hc)		6 (sc)		2 (sc)	4 (sc)	1 (sc)
9qh-	1 (hc)	2 (hc)		1 (sc)		8 (sc)	1 (sc)	
9cenh+		2 (hc)		1 (hc)		4	3	
9cenh-				1 (hc)				
inv(9)(var1)		2 (hc)			1			
inv(9)(var2)		1 (hc)	1 (hc)	1 (hc)				
9qh-9cenh+				2 (hc)				
9ph+9cenh+			1 (hc)					
9ph+9cenh+9qh-		1 (hc)						
inv(9)(var1)9ph+		1 (hc)						
dic(9)(var3)		1 (hc)				1 (hc)		

Using the 9het-mix four types of pericentric inversions, three types, each of dicentric variants and of size variants of 9ph or 9qh, as well as two types, each, of centromere-near duplications or size variations of the alpha-satellite region were detectable (Figure 1). The most frequently heteromorphisms observed were pericentric inversions (49.4%) followed by 9qh-variants (23.9%), variants of 9ph (11.4%), cenh (8.2%), and dicentric- (3.8%) and duplication-variants (3.3%) (Table 1).

Among the inversions types, inv(9)(var1) and inv(9)(var2) were most frequent, and constituted 48% and 47% of the inversion cases each (Tab. 1). These variants were previously reported as ‘inv (var1)’ and ‘inv (var2)’ in [6]. Besides, two yet unreported inversion types were detected: inv(9)(var2a) differing from inv(9)(var2) by a smaller and weaker midi36 positive signal in the short arm, and inv(9)(het). The latter variant was only seen once, and can also be described as inv(9)(p11.1q12) (Figure 1 and Table 3).

**Table 3:** **Suggestion for a new nomenclature of the 24 yet known chromosome 9 heteromorphisms: the name for the variant, the description according to ISCN (2009) [**[20]**] and if and where the variant was reported previously are given**

**Variant**	**ISCN description**	**Previously reported as [Ref]**
Found in the present study		
inv(9)(var1)	inv(9)(p12q21.11)	- inv(var1) [6]
inv(9)(var2)	inv(9)(p13.1q21.11)	- inv(var2) [6]
inv(9)(var2a)	inv(9)(p13.1q21.11)	- n.a.
inv(9)(het)	inv(9)(p11.1q12)	- n.a.
9qh+	9qh+ or amp(9)(q12)	- 9qh+ [28]
		- 9qh+ [6]
9qh+(var1)	amp(9)(q13q21.11)	- 9q21 amplification variant [9,10]
9qh-	9qh- or del(9)(q12)	- 9qh- [6]
9ph+	9ph+ or dup/trp(9)(p11.2~12)	- 9ph+ [6]
		- 9p12 duplication variant [9]
9ph++ (type 1)	9ph++ amp(9)(p11.2~12)	- 9p12 amplification variant [8,9]
9ph++ (type 1)		- In [7] two subtypes distinguished
		- 9p12 amplification variant [10]
9ph-	9ph- del(p11.2~12)	- 9qh- [29,30]
dic(9)(var1)	dic(9)(pter->q21.11::p13.1->qter)	- 9qh+ and inv (var5) [6]
dic(9)(var2)	dic(9)(pter->q12::p11.2->qter)	- n.a.
dic(9)(var3)	dic(9)(pter->q11::p11.1->qter)	- inversion type A [4]
dup(9)(var1)	der(9)(pter->q21.11::q12->qter)	- inversion type I or type III [2]
		- 9qh+ and inv (var3) [6]
		- extra signal(s) in var 9q12 [9]
		- 9q12 insertion variant [10]
dup(9)(var2)	der(9)(pter->q12::q21.11->q12::q21.11->q21.11::q12->qter)	- 9qh+ and inv (var4) [6]
9cenh+	9cenh+	- n.a.
9cenh-	9cenh-	- n.a.
Found in other studies		
inv dup(9)(var1)	der(9)(pter->q13~21.11::q13~21.11->p1?1.1::q13~21.11->qter)	- inversion type II [2] originally
		- reported only once [31]
dic(9)(var4)	inv(9)(p10q12)	- inversion type A [3]
		- inversion type D [4]
dic(9)(var5)	der(9)(pter->p10::q12->q11~12::q12->q11~12::q10->qter)	- inversion type C [3]
		- inversion type C [4]
inv(9)(het)(var1)	inv(9)(q11~12q12~13)	- inversion type B [3]
		- inversion type B [4]
del(9q)(var1)	del(9)(q13q21.11)	- 9q21 deletion variant [9,10]
		- also postulated to exist by [2]
trip(9)(var1)	der(9)(pter->q21.11::q12->q21.11::q12->qter)	- 9q12 euchromatic variant (EV) triplication
		- variant [9]

Size variants of the centromere-near heterochromatin in the long arm of chromosome 9 were described as either 9qh+ (87%) or 9qh- (13%) (Table 1). In two of the 9qh+ cases the size variant was not due to an enlarged midi18-positive band, but an enlargement of the midi36-postive band in 9q (Figure 1). This variant was classified as 9qh+(var1). Concerning centromere-near heterochromatin in the short arm of chromosome 9, 98% had a 9ph+ or 9ph++ and only one had a 9ph- (Table 2 and Figure 2).

Among the three dicentric variants of chromosome 9 (Table 1 and Figure 1) two were newly characterized in this study using the 9het-mix: dic(9)(var2), i.e. dic(9)(pter->q12::p11.2->qter) and dic(9)(var3), i.e. dic(9)(pter->q11::p11.1->qter). The most frequent variant dic(9)(var1) (13 out of 16 cases) was previously reported as ‘9qh+ and inv (var5)’ in [6].

The two duplication variants dup(9)(var1) and dup(9)(var2) (Table 1; Figure 1) were previously classified as ‘9qh+ and inv (var3)’ and ‘9qh+ and inv (var4)’, respectively [6].

**Figure 3:**
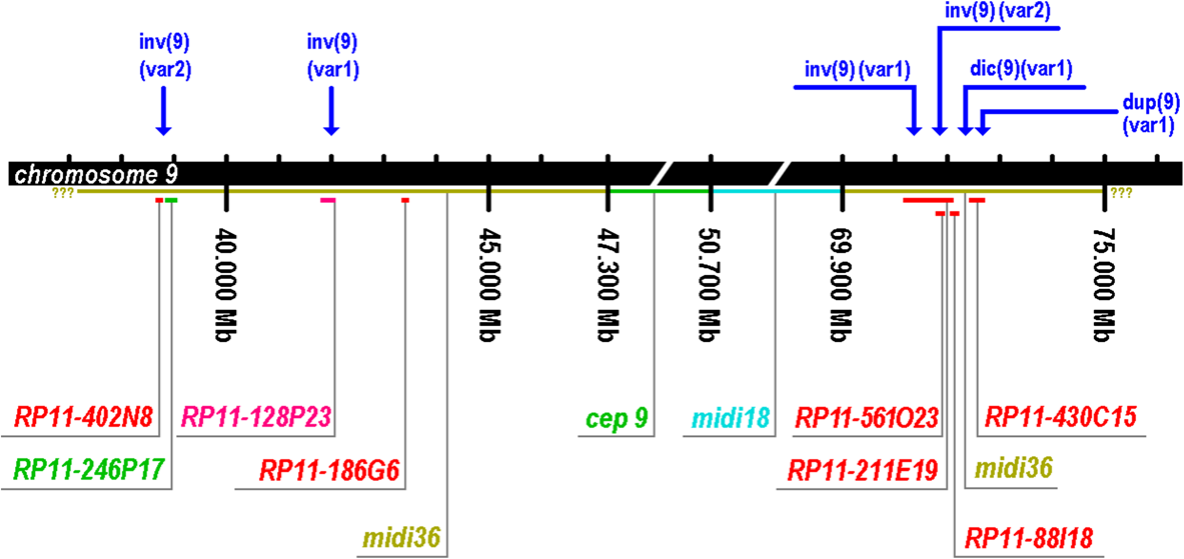
**Schematic depiction of the pericentric region of chromosome 9: small marks on top of the black bar symbolizing the chromosome indicate 10 Mb distances.** The regions of hybridization for probes midi18, midi36 and alpha satellite of chromosome 9 (cep 9) are indicated by the greenish, light-blue and green bars below the black line. Localizations of all applied BAC-probes are indicated by small red, green or pink bars below. Breakpoints of four of the studied variants, as determined by the FISH-results shown in Figure 3, are marked by blue arrows.

Twelve out of the 17 variants characterized by the 9het-mix were further studied applying two to eight of the BAC-probes (Figure 3). Five of the variants were not further analyzed by these probes since they showed no interest for this kind study (variants cenh+ and cenh-) or no material was available any more (variants 9ph++, dic(9)(var2) and dic(9)(var3)).

Applying these BAC-probes no further subgroups of those characterized by 9het-mix could be found in the cases studied.

Variants 9qh+ and 9qh- showed the same BAC-FISH patterns as the normal variant depicted in Figure 2 (FISH results not shown).

Results obtained for inv(9)(var1) indicated (Figure 2) that the corresponding breakpoints appeared within BAC RP11-128P23 in 9p12 and RP11-211E19 in 9q21.11,

Inv(9)(var2) as well as in inv(9)(var2a) the breakpoints lay with in the BACs RP11-402N8 in 9p13.1 and RP11-561O23 in 9q21.11 (Figure 2).

The inversion variant inv (9)(het) did only involve breakpoints proximal from RP11-186G6 in 9p11.2 and RP11-88I18 in 9q21.11, respectively (Figure 2).

In all the five cases of 9ph+ variant studied, amplification of RP11-402N8 and RP11-246P17 in 9p13.1 and of RP11-128P23 in 9p12 could be observed (Figure 2). Also RP11-211E19 in 9q21.11, RP11-561O23 in 9q21.11 and RP11-88I18 in 9q21.11 gave strong cross hybridization signals in the 9ph+ region.

In the 9ph- case the signals for RP11-128P23 in 9p12 were weaker than normally (Figure 2). Strikingly, also the cross hybridization signal of RP11-561O23 in 9p12 was almost not visible in this case.

The 9qh+(var1) showed amplification of all tested BAC probes besides RP11-430C15 in 9q21.11. Not only BACs in 9q21.11 (RP11-561O23, RP11-88I18, RP11-430C15) but also BACs in 9p (RP11-402N8, RP11-128P23) showed strong (cross-) hybridization in 9q21.11. Nonetheless most likely there is an amplification of 9q21.11 material, only (Figure 2).

For the heteromorphic pattern described as dic(9)(var1) BAC-FISH confirmed that there is a dic(9)(pter->q21.11::p13.1->qter). The breakpoint in 9p is distal from BAC RP11-246P17 in 9p13.1; the break in 9q21.11 appeared between the BAC probes RP11-561O23 and RP11-88I18 (Figure 2).

A duplication within the long arm of chromosome 9 is present in variant dup(9)(var1) according to BAC-FISH results. According to BAC-FISH, one of the breakpoints in 9q is spanned by RP11-88I18 in 9q21.11, as the ‘central’ of the three obtained signals is weaker than expected. Thus, dup(9)(var1) can be described as a der(9)(pter->q21.11::q12->qter).

For dup(9)(var2) BAC-FISH could only confirm that the duplication appeared in 9q. However, we speculate, that dup(9)(var2) evolved by a paracentric inversion in 9q12 out of dup(9)(var1), which could be described as der(9)(pter->q12::q21.11->q12::q21.11->q21.11::q12->qter).

All BAC-results are schematically summarized in Figure 2.

Indications for cytogenetic diagnostics were in a comparable range for infertility, DD/MR and others in Eastern and Western Europe. However, as in Armenia and Belarus, prenatal diagnostics is not offered on regularly bases as in Western Europe, thus heteromorphic variants were detected less frequently in this group of patients. Interestingly, heterochromatic variants were found 1.4 times more frequently in female compared to male (Tab. 1).

Parental origin of the heteromorphisms was mainly studied in case of DD/MR in the index patient. Hereby maternal origin was detected 1.3 times more frequently than paternal one (Tab. 1). Interestingly, in one family, in two subsequent pregnancies a male and a female fetus had inherited the inv(9)(var1) from the mother and the 9qh+ from the father. Only in one case a de novo origin was detectable for an inversion type inv(9)(var1), appearing on a maternal chromosome 9, identifiable due to a 9ph+ variant; the second, paternal chromosome 9 had a 9qh+(pat).

Comparing the seventeen identified heteromorphic pattern of this study with others previously reported in the literature, twelve were already reported by us or others before (Table 3). Besides, five new variants (inv(9)(var2a), inv(9)(het), dic(9)(var2), 9cenh+, 9cenh-) are presented in this study. A review of the literature identified seven additional heteromorphic patterns (9ph++ type 1 or 2, inv dup(9)(var1), dic(9)(var4), dic(9)(var 5), inv(9)(het)(var1), del(9q)(var1) and trip(9)(var1) (Table 3). As four different names are already suggested per variant so far, we suggest a nomenclature for the overall 24 yet reported heteromorphic patterns, which may easily be enlarged in case of new variants described (Table 3).

## Discussion

To the best of our knowledge this is the largest study ever done in the carriers of chromosome 9 heteromorphisms. The samples from all the 334 patients were collected over ~10 years in Western Europe (one laboratory in Jena, Germany) and one laboratory in Belarus (Minsk), Turkey (Ankara) and Armenia (Yerevan) (i.e. Eastern Europe). In this study, 17 different heterochromatic variants were identified using the 9het-mix (Figure 1). According to literature (Table 3) the most frequent variants are covered by that probe set. Among the five new variants there are also two, which are well-known already: i.e. size variations of the alpha-satellite region of chromosome 9 (cenh+ and cenh-); however, these were not reported in the literature yet. Nonetheless, they have to be considered as well as important in chromosome 9 heteromorphisms, as it is known that this kind of variant may also change the GTG-banding pattern [19].

The further three new variants (inv(9)(var2a), inv(9)(het), dic(9)(var2)) were newly discovered in this study and were to the best of our knowledge not reported previously. As summarized in Tab. 3 six further variants of pericentric heterochromatin of chromosome 9 were reported by others before. Among them inv dup(9)(var1), dic(9)(var4), dic(9)(var5), trip(9)(var1) and del(9q)(var1) could principally be detected by 9het-mix. However, neither dic(9)(var4) and dic(9)(var5) nor 9ph++ type 1 and type 2 could be distinguished from each other. The variant inv(9)(het)(var1) could only be identified if a chromosome 9-specific ß-satellite probe were applied [3,4].

24 molecular cytogenetic variants of chromosome 9 heteromorphisms were reported so far. 21 of them can be identified and characterized by the 9het-mix. As summarized in Tab. 3 up to four different names are already suggested per variant; thus, a new nomenclature for 24 heteromorphic patterns already known might be helpful (Tab. 3). This nomenclature is based on ISCN (2009) [20], which does not provide a corresponding nomenclature yet. Table 3 also provides a classical banding cytogenetic description of each variant not applying the “ish-nomenclature” emphasized by ISCN (2009).

According to our results it can be stated that pericentric inversions are more frequent among the heteromorphic patterns of chromosome 9 (49.4%). Size variations of 9qh, 9ph and the centromeric region constitute together the second largest group (43.5%), while dicentrics and duplication- variants are rare. The same might be true for the variants not seen in this study, such as inv dup(9)(var1), dic(9)(var4) or dic(9)(var 5), del(9q)(var1) or trip(9)(var1).

Additionally, the 24 observed pattern may be present in combination with each other as well. As summarized in Tab. 2, in 43 of the 334 cases two or even three variants were present altogether on the same chromosome. Even up to four or more different patterns may be observed in one patient if two chromosomes 9 with different heteromorphic patterns come together. Such instances can also be found in the corresponding literature [6].

In 11 of the 334 cases studied (3.3%) there were additional chromosomal aberrations besides the chromosome 9 heteromorphisms. This rate is in the range of normal rate of chromosomal aberrations in the general population [21].

The pattern of 9qh+(var1) was found only twice in this study. One patient originated from Greece and the second one from Korea. Interestingly, recently it has been reported that this variant was observed 2.4 times in 1000 recurrent births specifically in Korea [22]. For the seventeen variants studied here, in general, no difference in the appearance frequencies for Eastern and Western Europe was observed. The variants 9ph+ dic(9)(var1) and dup(9)(var1) were exceptions. All the three of them were observed 5.3, 7.0 and 3 times more frequently in Western compared to Eastern Europe. A founder effects for these variants might be considered.

Some of the heteromorphic patterns of chromosome 9 were already studied in more detail using BAC-probes. In this study, BACs applied by others and in our own previous studies, were used to narrow down some of the breakpoints involved in the heteromorphic rearrangements of chromosome 9 [7-10,18]. As summarized in Figure 3 the breakpoints for inv(9)(var1), inv(9)(var2), dic(9)(var1) and dup(9)(var1) were in parts close to each other, in the q-arm in the range of less than 20 Mb. However, they neither co-localize nor overlapped. Thus, it might be suggested that the pericentric region of chromosome 9 is somehow ‘breakpoint prone’ [23] due to segmental duplications [10]. It can be speculated that at least some of the variants can be deduced on a unique event, like those mentioned in the previous paragraph: 9qh+(var1), 9ph+ dic(9)(var1) and dup(9)(var1). Also, it would be interesting to study acquired inversion-9-variants in human cancer, which were repeatedly seen (e.g. [24]).

The influence of heteromorphic patterns of chromosome 9 on infertility is repeatedly discussed [6; 11–16]. Almost 30% of the carriers of chromosome 9 variants studied here were referred due to infertility. Among this group most frequently found were inv(9)(var2) (27%), inv(9)(var1) (22%), 9qh+ (18%), 9ph+ (10%), 9qh- (7%), 9cenh+ (6%) and dup(9)(var1) (4%). These values can be aligned nicely with the general frequencies of these kinds of heteromorphisms; thus, according to this study there is no evidence for a correlation of any of the chromosome 9 heteromorphic patterns with infertility. Also, at least for four of the infertility patients of the present study, a parental origin of the chromosome 9 variant was proven. Maybe similarly as in patients with infertility and a small supernumerary marker chromosome (sSMC) there is, at least in parts, a yet unknown reason for an ascertainment bias: ~50% of infertile sSMC patients inherited the sSMC by one of their parents [25]. While for sSMC patients a selection against the marker chromosome is present in the male germ line [26] a similar effect is not definitely proven for heteromorphisms of chromosome 9 [11,14-16]. In this study, for 39 patients the parental origin could be determined. Maternal origin was observed in 56%, paternal origin in 44%, i.e. 1.3 times more frequently, and even a de novo event was proven in one case. According to this data there could be a slight selection against heteromorphic patterns of chromosome 9 via the paternal line; however, this suggestion would need to be checked on more cases. Also together with the aforementioned data that there is no hint on a different distribution of chromosome 9 variants in infertile compared to all studied patients, the 56% to 44% rate could also be interpreted as “almost 1:1”. The latter would also be supported by the fact that most of the variants of chromosome 9, if tested, passed through one or more previous generations already [8,27]. Still it remains unclear why, in the present study, heterochromatic chromosome 9 variants were found 1.4 times more frequently in female compared to male (Table 1).

## Conclusion

In summary, chromosome 9 heteromorphisms turned out to be more complex than initially suggested. They may be best detected by means of banding cytogenetics, and further characterized by molecular cytogenetics, i.e. FISH. Array-comparative genomic hybridization (aCGH) may run into problems analyzing such variants; i.e. the pericentric region of chromosome 9 is not well covered in most aCGH settings. Either this approach misses the variants entirely [22] or it delivers ambiguous results with false positive signals along chromosome 9 and other genomic regions [10].

## Competing interests

The author(s) declare that they have no competing interests.

## Authors' contributions

This study was carried out over more than 10 years and was only possible due to the collection of many cases. MMa, IS, HM, RA, ADP, AIK, TE, EJ, AE, NS, IVM, MV, IS, HN, MS, R-DW, GR-A, Mme, LB, TM, LR, AW, AS, SV and EE provided cases and/or did primary cytogenetic tests; TL planned the studies and drafted the paper together with MdBC; NK, AG, MMa, HM, ADP, AIK, Mme, SB, MZ, KK and EE did detailed FISH studies. All authors read and approved the final manuscript.
